# Supplementary material for: A tree of leaves: Phylogeny and historical biogeography of the leaf insects (Phasmatodea: Phylliidae)
Source: Commun Biol. 2021 Aug 2;4:932. doi: 10.1038/s42003-021-02436-z (PMC8329230; doi:10.1038/s42003-021-02436-z)
Supplement: Supplementary file 1 — Reporting Summary [file 42003_2021_2436_MOESM1_ESM.pdf]

nature research

Corresponding author(s): Sarah Bank, Royce T. Cumming

Last updated by author(s): Jun 22, 2021

Reporting Summary

Nature Research wishes to improve the reproducibility of the work that we publish. This form provides structure for consistency and transparency in reporting. For further information on Nature Research policies, see our Editorial Policies and the Editorial Policy Checklist.

Statistics

For all statistical analyses, confirm that the following items are present in the figure legend, table legend, main text, or Methods section.

n/a	Confirmed	
☒	☐	The exact sample size (n) for each experimental group/condition, given as a discrete number and unit of measurement
☒	☐	A statement on whether measurements were taken from distinct samples or whether the same sample was measured repeatedly
☒	☐	The statistical test(s) used AND whether they are one- or two-sided<br>*Only common tests should be described solely by name; describe more complex techniques in the Methods section.*
☒	☐	A description of all covariates tested
☒	☐	A description of any assumptions or corrections, such as tests of normality and adjustment for multiple comparisons
☒	☐	A full description of the statistical parameters including central tendency (e.g. means) or other basic estimates (e.g. regression coefficient) AND variation (e.g. standard deviation) or associated estimates of uncertainty (e.g. confidence intervals)
☒	☐	For null hypothesis testing, the test statistic (e.g. F, t, r) with confidence intervals, effect sizes, degrees of freedom and P value noted<br>*Give P values as exact values whenever suitable.*
☐	☒	For Bayesian analysis, information on the choice of priors and Markov chain Monte Carlo settings
☒	☐	For hierarchical and complex designs, identification of the appropriate level for tests and full reporting of outcomes
☒	☐	Estimates of effect sizes (e.g. Cohen's d, Pearson's r), indicating how they were calculated

Our web collection on statistics for biologists contains articles on many of the points above.

Software and code

Policy information about availability of computer code

Data collection	no software was used to collect data
Data analysis	Geneious v.11.0.5, mafft v.7.450, MACSE v.2.03, FASCONCAT v.1.1, IQ-TREE v.2.1.1, RAxML v.8.2.12, FigTree v.1.4.4, BEAST v.2.6.1, Tracer v.1.7.1, BioGeoBEARS v.1.1.2, R 3.5.3, bPTP web server

For manuscripts utilizing custom algorithms or software that are central to the research but not yet described in published literature, software must be made available to editors and reviewers. We strongly encourage code deposition in a community repository (e.g. GitHub). See the Nature Research guidelines for submitting code & software for further information.

Data

Policy information about availability of data

All manuscripts must include a data availability statement. This statement should provide the following information, where applicable:

- Accession codes, unique identifiers, or web links for publicly available datasets
- A list of figures that have associated raw data
- A description of any restrictions on data availability

Newly generated sequence data were deposited in GenBank under the accession numbers MW686032–MW686200, MW698871–MW698927, MW703187–MW703369. The authors declare that the data supporting the findings of this study are available within the supplementary information files.

Field-specific reporting

Please select the one below that is the best fit for your research. If you are not sure, read the appropriate sections before making your selection.

☐ Life sciences ☐ Behavioural & social sciences ☒ Ecological, evolutionary & environmental sciences

For a reference copy of the document with all sections, see nature.com/documents/nr-reporting-summary-flat.pdf

Ecological, evolutionary & environmental sciences study design

All studies must disclose on these points even when the disclosure is negative.

Study description	Phylogenetic analysis of the leaf insects with divergence times and ancestral range estimation based on DNA sequence, fossil and geographical data.
Research sample	We used 96 representatives of leaf insects (Phylliidae) and included 73 outgroup species of other phasmatodean lineages. The taxon sampling covers all genera and major groups, and the majority of described species plus at least 15 undescribed and new species of leaf insects.
Sampling strategy	We sampled all available specimens for our phylogenetic and biogeographical analyses, except for four species whose sequence data was not yet available at the time of our analysis (Cryptophyllium khmer, C. liyananae, C. nuichuaense, C. wennae; these are however included in our publication in ZooKeys, doi: 10.3897/zookeys.1018.61033)
Data collection	Sequence data was "collected" by sequencing the DNA of the specimens.
Timing and spatial scale	na
Data exclusions	no data was excluded, but the amplification of the 12S gene with primers used in previous studies on Phasmatodea was omitted because of a low success rate (in comparison to other phasmatodean groups)
Reproducibility	na
Randomization	na
Blinding	na

Did the study involve field work? ☐ Yes ☒ No

Reporting for specific materials, systems and methods

We require information from authors about some types of materials, experimental systems and methods used in many studies. Here, indicate whether each material, system or method listed is relevant to your study. If you are not sure if a list item applies to your research, read the appropriate section before selecting a response.

Materials & experimental systems

n/a	Involved in the study	
☒	☐	Antibodies
☒	☐	Eukaryotic cell lines
☒	☐	Palaeontology and archaeology
☒	☐	Animals and other organisms
☒	☐	Human research participants
☒	☐	Clinical data
☒	☐	Dual use research of concern

Methods

n/a	Involved in the study	
☒	☐	ChIP-seq
☒	☐	Flow cytometry
☒	☐	MRI-based neuroimaging
