# Supplementary material for: A tree of leaves: Phylogeny and historical biogeography of the leaf insects (Phasmatodea: Phylliidae)
Source: Commun Biol. 2021 Aug 2;4:932. doi: 10.1038/s42003-021-02436-z (PMC8329230; doi:10.1038/s42003-021-02436-z)
Supplement: Supplementary file 2 — Supplementary Information [file 42003_2021_2436_MOESM2_ESM.pdf]

SUPPLEMENTARY INFORMATION

for

A tree of leaves: Phylogeny and historical biogeography of the leaf insects (Phasmatodea: Phylliidae)

Sarah Bank*, Royce T. Cumming*, Yunchang Li, Katharina Henze, Stéphane Le Tirant and Sven Bradler

Corresponding authors:

Sarah Bank (sbank.bio@gmail.com) and Royce T. Cumming (phylliidae.walkingleaf@gmail.com)

Table of Contents

Supplementary Discussion

A. Combined morphological and molecular results

B. Generic differentiation of Phylliidae

C. Additional taxonomic acts

Supplementary Note 1

List and differentiation of phylliid genera

Supplementary Note 2

Keys to Phylliidae genera

Supplementary References

Supplementary Figures

Supplementary Figure 1

Supplementary Figure 2

Supplementary Figure 3

Supplementary Figure 4

Supplementary Figure 5

Supplementary Figure 6

Supplementary Discussion

A. Combined morphological and molecular results

In leaf insects (Phylliidae), it has proven difficult to base a phylogenetic hypothesis on morphology alone as morphological comparisons of one feature may link genera upon preliminary review, but as soon as a different feature is considered, different genera appear to be more prominently linked. Although the intergeneric relationships recovered by our molecular analysis when compared with morphological evaluations do thus not readily agree, we have found strong association of intrageneric molecular and morphological agreement within multiple clades. Hence, morphological characters substantiate the placement of species within the distinct clades (genera) as recovered by molecular analysis (see Supplementary Note 1 below for features, which clearly distinguish genera from each other) as well as in several instances the clearly supported interspecies relationships.

One reoccurring example is the relationship between species from Borneo and Palawan (yellow in Figures 5, 6). Two independent *Phyllium* clades have been found to be linked to this region where the resulting species have maintained morphological similarity despite notable molecular distances. One clade is comprised of *Ph. gantungense* (Palawan), *Ph. arthurchungi* (Borneo) and *Ph. cummingi* (Borneo), which exhibits a unique egg shape, distinct from all other phylliids, as the lateral surface is marked by eight prominent pits arranged in a two by four pattern. Adult morphology has also been relatively maintained as these species have males with distinctly long tegmina and both sexes have broad boxy abdominal shapes with the lobes on the terminal segments variable[1]. Interestingly, the Palawan clade females have conspicuous black coxae markings (a somewhat rare feature within the phylliids) versus their Bornean counterparts, which are the more common orange. Another clade from this area is formed by *Ph. palawanense* (Palawan) and *Ph. chenqiae* (Borneo). Although they seem to have diverged ~15 mya (Figure 5) they are morphologically unique with small sized and strongly tapered bodies and both sexes with the granulation along the prescutum filling the prescutum surface (which is uncommon as most phylliids have the granulation/spines along the sagittal plane).

Wallacea also presents a set of *Phyllium* species, which are morphologically well-linked and were recovered as one distinct clade in our analyses: *Ph. siccifolium* (Ambon, Buru, Seram), *Ph. mamasaense* (Sulawesi) and *Ph. letiranti* (Peleng, Sanana). The former two also share the black colouration of the coxae but are geographically separated by Weber's Line[2], and thus can be easily distinguished from *Ph. letiranti*, which is found with *Ph. mamasaense* west of the line. The general morphology of these three species all matches well with similar broad abdominal shapes, mesopleurae that are armed with prominent tubercle-like spines and prominently rounded, broad exterior profemoral lobes. Additionally, it is also known that *Ph. siccifolium* and *Ph. letiranti* show high intraspecific variety in regard to overall body colouration, often found as green, yellow or brown, whereas we can only speculate that this is also true for the poorly known *Ph. mamasaense*.

A final clear example to bring forward linkage of morphological and molecular results is the *Phyllium* clade consisting of *Ph. hausleithneri* (West Malaysia), *Ph. nisus* (Sumatra), *Ph. gardabagusi* (Java) and *Ph. jacobsoni* (Java). Before these four species were revealed to be distinct lineages[3], they had been considered as only two (based on white and purple coxae colouration) with a wide geographic distribution, significant intraspecific morphological variation found in all populations such as abdominal shapes ranging from smooth to strongly lobed[4]. Interestingly, the two species that occur sympatrically have differing coxae colouration: purple in *Ph. gardabagusi* and white in *Ph. jacobsoni*. The recurrent occurrence of closely related *Phyllium* species with similar overall morphology but distinctly differing coxae colouration (particularly in sympatry or geographically close areas) leads us to wonder if coxae colouration plays a role in sexual selection. However, Cumming et al.[3] had already hypothesised that these colourful displays might be a defensive strategy and possibly part of a startle display.

B. Generic differentiation of Phylliidae

Traditionally, Phylliidae contained only few genera with single species thought to inhabit thousands of kilometres of diverse habitat and span across seas[5,6]. Newly recognised taxa were described as subgenera rather than given full genus rank, a tendency that likely originated with Griffini[7], who described *Pulchriphyllium* as a subgenus of *Phyllium* despite the morphological disparity. More recently, extensive morphological studies started to illuminate the largely underestimated phylliid diversity, and yet, were still influenced by the traditional low-genera diversity mentality, which resulted in an overinflated *Phyllium* with numerous subgroupings[8–10]. These morphologically distinguishable subgroups were found to correspond to monophyletic clades in our molecular analysis, however, as drastically unrelated groups that demonstrated *Phyllium* as a paraphyletic lineage.

Based on our molecular phylogeny (Figure 2), two taxonomic options for a reclassification within the phylliids were considered. One option was to follow the historic view based upon the oldest recognised genera *Phyllium*, *Chitoniscus* and *Nanophyllium*, and to treat them as vastly diverse with many morphologically distinct subgenera within them. In contrast to past authors who believed Phylliidae to be a species-poor taxon (e.g., <20 species[5]), we now know that species diversity had in fact been highly underestimated due to overestimation of ranges, cryptic species diversity and a high amount of endemism in relation to isolated islands or along gradients of elevation. With over 100 species at present and increasing significantly every year, the number of described species has doubled in less than a decade[11,12]. Consequently, we chose to emphasise on the diversity of these lineages and aimed to clarify some of the taxonomic confusion within Phylliidae: With the inclusion of the nominate *Phyllium* type species *Ph. siccifolium* (Linnaeus, 1758) as well as the type species of all *Phyllium* subgenera in our molecular analysis, we were able to confidently render *Phyllium* monophyletic by elevating its well-supported subgenera to independent genera and thus reflecting their uniqueness from *Phyllium* sensu stricto.

The elevation to full genus rank of the three monophyletic clades, namely, *Pulchriphyllium* Griffini, 1898 **stat. rev.**, *Comptaphyllium* Cumming et al., 2019 **stat. rev.** and *Walaphyllium* Cumming et al., 2020 **stat. rev.**, created numerous novel binomial combinations (see Supplementary Data 1, 2) and an overview of the differentiation of all phylliid genera is given below (Supplementary Note 1). For a more detailed discussion on the morphological uniqueness of the newly elevated genera, please refer to Cumming et al.[9] for *Comptaphyllium*, Cumming et al.[10] for *Walaphyllium*, and Hennemann et al.[8] for a morphological review of *Pulchriphyllium*.

C. Additional taxonomic acts

In addition to the elevation of the *Phyllium* subgenera, the results of our analyses and the review of all relevant morphological studies allowed us to make some other significant taxonomic changes. *Phyllium brevipenne* Grösser, 1992, which was believed to belong to *Pulchriphyllium*, was recovered within the *Nanophyllium* clade. Further review of morphology confirmed that features such as the width/length ratio of the thorax and two lobed posteriomedial tubercle on the head capsule agree with this placement. Therefore, the new binomial is *Nanophyllium brevipenne* (Grösser, 1992) **comb. nov.** Within the true *Pulchriphyllium*, the Sri Lankan population was recovered as a full species, not a synonym or a subspecies of *Pu. bioculatum* (Gray, 1832). Therefore, this population is returned to its original status as a species, but is now contained within *Pulchriphyllium* **stat. rev.**, therefore the new binomial is *Pulchriphyllium agathyrsus* (Gray, 1843) **stat. rev.**, **comb. nov.** Future studies will need to sample the other populations of *Pu. bioculatum* to clarify their statuses as subspecies or full species.

Several surprises revealed themselves within the clade comprising *Microphyllium* Zompro, 2001 and *Pseudomicrophyllium* Cumming, 2017, but subsequent morphological review appeared to further substantiate the molecular results. First, *Pseudomicrophyllium faulkneri* Cumming, 2017 was unveiled as the male counterpart to *Microphyllium pusillulum* Rehn & Rehn, 1934, which prompted us to further review the validity of the two genera. While male *Microphyllium* have antennae, which are about as long as their extended forelegs and are composed of short bead-like segments, and profemoral interior lobes

that are narrow and marked with three distinct teeth, *Pseudomicrophyllium* males exhibit the more typical phylliid antennae that are long and thin and profemoral interior lobes reduced to only a single anterior spine. Female *Microphyllium*, whose gonapophyses are notably short (only slightly protruding from under the subgenital plate), have the middle most spine along the prescutum as the largest spine with the posterior most spine highly reduced. In contrast, *Pseudomicrophyllium* females have gonapophyses that are long (reaching to the apex of the anal abdominal segment) and their posterior most spine of the prescutum is the most prominent. In consideration of these significantly differing morphologies and a relatively old divergence time for the split of *Microphyllium* and *Pseudomicrophyllium*, we have decided to treat the two genera as independent instead of merging them. This implies that even though *Ps. faulkneri* is a synonym of *M. pusillulum*, this species is transferred from *Microphyllium* to form the new binomial *Pseudomicrophyllium pusillulum* (Rehn & Rehn, 1934) **comb. nov.** (now known from both male and female specimens). This leaves only *M. spinithorax* Zompro, 2001 and *M. haskelli* Cumming, 2017 within *Microphyllium* sensu stricto. The other surprising finding among *Pseudomicrophyllium* was that *Phyllium geryon* Gray, 1843 was recovered as sister to *Ps. pusillulum*. Interestingly, these two sister species are significantly different in size, but when size is ignored, the features of the thorax and genitalia place this species unambiguously within *Pseudomicrophyllium*. We additionally reviewed the little-known eggs of this genus. One live female of *Ps. pusillulum* was collected by Thierry Heitzmann in 2010 and was kept alive for a few days to lay eggs. Interestingly, these eggs were small and spherical and thus quite unique for Phylliidae. When we dissected the female *geryon* specimen that was used for the molecular analysis, we found that her eggs were in fact matching the egg morphology of *Ps. pusillulum*. Therefore, based on both molecular and morphological evidence, *Phyllium geryon* is transferred to create the novel binomial *Pseudomicrophyllium geryon* (Gray, 1843) **comb. nov.** This leaves both of these rarely collected and morphologically quite unique genera with two species each, but we are aware of several additional records from throughout the Philippines in need of further review in order to further understand this clade's diversity.

As was found in other past studies[13–16], *Chitoniscus* Stål, 1875 was recovered as paraphyletic, but our sampling of this poorly known group lacked a confidently identified specimen of the *Chitoniscus* type species *Ch. lobiventris* Blanchard, 1853 and our overall taxon sampling was limited/based upon immature specimens which could not be identified to species. Therefore, a taxonomic adjustment of this group must be withheld at this time until more sampling can be done to clarify the species of this region.

Supplementary Note 1

List and differentiation of phylliid genera

***Phyllium* Illiger, 1798**

Type species: *Gryllus siccifolius* Linnaeus, 1758 (= *Phyllium siccifolium* (Linnaeus, 1758)) by original monotypy

Differentiation:

Female: Tegmina with media and cubitus veins distinctly separated with several vein widths distance between them throughout the length, not touching; prescutum which is the same width as length, or notably longer than wide; tegmina cubitus venation simple (unsplit) or bifurcate (into an anterior cubitus (CuA) and posterior cubitus (CuP1) only); tibiae lacking exterior lobes; mesopleura anterior the same width as the anterior of the prescutum, not notably broader.

Male: Tibiae lacking exterior lobes; profemoral interior lobe broader than the exterior lobe; mesopleura anterior the same width as the anterior of the prescutum, not notably broader.

Egg: Surface with pinnae (rope- or feather-like); operculum with pinnae encircling the margin, not along the sagittal plane.

Fresh Nymph: Base colouration typically black or dark brown; occasionally reddish colouration as the base colour on the femora; pro-, meso-, metafemora typically with a white or yellow stripe fully or partially crossing the leg; abdomen can have some green colouration on the margins throughout, or typically is mostly black/brown.

***Pulchriphyllium* Griffini, 1898**

Pulchriphyllium Griffini, 1898 **stat. nov.**, raised to genus rank.

Type species: *Phyllium pulchrifolium* Audinet-Serville, 1838 (= *Pulchriphyllium pulchrifolium* (Audinet-Serville, 1838)) by original designation

Differentiation:

Female: Tegmina cubitus venation simple (unsplit) or bifurcate (into an anterior cubitus (CuA) and posterior cubitus (CuP1) only); tegmina with media and cubitus veins running side by side and touching throughout the majority of their length; tibiae with well-developed exterior lobes; prescutum which is the same width as length, or notably longer than wide; terminal antennomere as long as the preceding one or two segments combined.

Male: Alae radial sector, media anterior, and media posterior veins fusing to the cubitus at different locations along the vein and running together to the wing margin; vomer with a single apical hook; protibiae always with a fully developed interior lobe; meso- and metatibiae exteriors with fully developed lobes.

Egg: Lacks pinnae; surface brittle/stiffly spongey; Lateral margins fanned out into distinct fins with an operculum which is typically longer than wide (but not always), or if the capsule fins are reduced (not prominently protruding), the egg in cross-section is distinctly triangular (not pentagonal or rectangular), with the dorsal surface notable broader than the other surfaces.

Fresh Nymph: Exterior profemoral lobe prominent; meso- and metatibiae with notable exterior lobes; abdomen broad (abdominal length less than two times the width); overall colouration red, brown, orange.

Walaphyllium Cumming, Thurman, Youngdale & Le Tirant 2020

Walaphyllium Cumming, Thurman, Youngdale & Le Tirant, 2020 **stat. nov.**, raised to genus rank.

Type species: *Phyllium zomproi* Grösser, 2001 (=*Walaphyllium zomproi* (Grösser, 2001)) by original designation

Differentiation:

Female: Tegmina venation with the posterior cubitus split into an anterior cubitus (CuA), first posterior cubitus (CuP1), and second posterior cubitus (CuP2); tibiae lacking exterior lobes; fourth antennal segment about as long as the following segment individually, not short disk-like; lack developed alae.

Male: Abdominal shape rectangular, with segments V and VI with fully parallel-sided margins (segments IV and VII with only half parallel-sided and the remainder curved); tegmina media vein with an anterior media vein (MA) and two posterior media veins (MP1 and MP2); vomer with a single apical hook

Egg: Capsule lacking pinnae, instead with a brittle sponge-textured surface and the operculum conically raised.

Fresh Nymph: (Only known for *monteithi*) Slender legs and abdomen; base colouration brown, front legs orange, margins of anterior abdominal segments mint green; white striping on the meso- and metafemoral lobes.

Comptaphyllium Cumming, Le Tirant & Hennemann, 2019

Comptaphyllium Cumming, Le Tirant & Hennemann, 2019 **stat. nov.**, raised to genus rank.

Type species: *Phyllium caudatum* Redtenbacher, 1906 (=*Comptaphyllium caudatum* (Redtenbacher, 1906)) by original designation

Differentiation:

Female: Protibial interior lobe not reaching from end to end of the shaft, only restricted to the proximal half to two-thirds but never more; head capsule with clearly defined nodes arranged in evenly spaced patterns; anterior rim of the prescutum with a large prominent spine; prosternum with a prominent swelling with a granular surface; antennae which are long and slender (apical antennomere IX as long as or longer than the proceeding three segments combined); alae which are small but present (5-10 mm in length); mesopleura which have four of five short rounded tubercles; terminal segments of the abdomen always significantly tapered more than the preceding segments giving the abdomen a clear spade shaped appearance.

Male: Protibial interior lobe not reaching from end to end of the shaft, only restricted to the proximal half to two-thirds but never more; head capsule with clearly defined nodes arranged in evenly spaced patterns; anterior rim of the prescutum with a large prominent spine; prosternum with a prominent swelling with a granular surface; abdomen long and narrow (only about 30% as wide as long); mesopleura with five well defined spines; tegmina short, only reaching the anterior margin of abdominal segment III.

Egg: With long frill like pinnae along the lateral margins on three margins giving the egg a triangular cross-section; operculum with a medial row of pinnae along the sagittal plane, not pinnae encircling the margin of the operculum.

Fresh Nymph: (Only known for *caudatum*) Slender legs and abdomen; base colouration brown throughout; metafemoral lobes with a prominent lime-green stripe.

***Cryptophyllium* Cumming, Bank, Bresseel, Constant, Le Tirant, Dong, Sonet & Bradler, 2021**

Type Species: *Phyllium celebicum* de Haan, 1842 (=*Cryptophyllium celebicum* (de Haan, 1842)) by original designation

Differentiation:

Female: Antennae with nine segments of which the fourth antennal segment is short and disk-like; can have highly reduced alae (rare) or more commonly decently developed alae which can be half to almost the same length as the tegmina; interior tibial lobes present on the protibiae and some species either have full exterior meso-, and metatibial exterior lobes; lobes on the pro-, meso-, and metatibial exterior, which are small and reduced to the distal end only; or have meso-, and metatibiae, which are completely devoid of exterior lobes.

Male: Vomer with two apical hooks; prescutum longer than wide; interior tibial lobes on the protibiae and some species either have full exterior meso-, and metatibial exterior lobes; lobes on the pro-, meso-, and metati- bial exterior, which are small and reduced to the distal end only; or have meso-, and metatibiae, which are completely devoid of exterior lobes.

Egg: Without strongly formed lateral fins; instead, the eggs are rather boxy in appearance with relatively straight margins and short moss-like pinnae on most surfaces; the egg operculum is conically raised and shares a similar surface texture to the overall egg with short moss-like pinnae throughout, but typically with slightly longer pinnae along the margins of the operculum.

Fresh Nymph: The exterior profemora with a distinct lobe; typically the body has brown, black, or red base colouration; meso- and metafemoral lobes typically with a white stripe crossing, occasionally the profemoral also has a white stripe; abdominal segments II-IV typically with prominent green spots.

***Microphyllium* Zompro, 2001**

Type Species: *Microphyllium spinithorax* Zompro, 2001 by original designation

Differentiation:

Female: Middle most spine along the prescutum is the largest spine and the posterior most spine is highly reduced; gonapophyses notably short, only slightly protruding from under the subgenital plate; third antennomere lacking stridulantory file.

Male: Antennae which are about as long as extended forelegs and are composed of short bead-like segments; profemoral interior lobes which are narrow and marked with three distinct teeth; less than 30 mm long.

Egg: Unknown.

Fresh Nymph: Unknown.

***Pseudomicrophyllium* Cumming, 2017**

Type Species: *Pseudomicrophyllium faulkneri* Cumming, 2017 (= *Phyllium pusillulum* Rehn & Rehn, 1934, = *Pseudomicrophyllium pusillulum* (Rehn & Rehn, 1934)) by original designation

Differentiation:

Female: Posterior most spine of the prescutum is the most prominent; gonapophyses which are long, reaching to the apex of the anal abdominal segment.

Male: Antennae which are long and thin; profemoral interior lobes reduced to only a single anterior spine; less than 30 mm long.

Egg: Small and spherical; no pinnae.

Fresh Nymph: Unknown.

***Chitoniscus* Stål, 1875**

Type Species: *Phyllium lobiventre* Blanchard, 1853 (=*Chitoniscus lobiventris* (Blanchard, 1853)) by original monotypy

Differentiation:

Female: Protibial interior lobes variable, can be absent, only on the proximal half, or well developed; tegmina with media and cubitus veins running side by side, touching throughout the majority of their lengths or fused throughout most of their length.

Male: Prescutum stout, ca. 2× as wide as long; protibial interior lobes variable, can be absent, only on the proximal half, or well-developed.

Egg: Lack pinnae; rectangular eggs; surface with granulation/sparse pitting.

Fresh Nymph: (Only known for *sarrameaensis*) body and legs slender; base colouration brown; white striping on the meso- and metafemoral lobes, not fully crossing the leg (mostly just on the exterior lobe and femoral shaft); margins of abdominal segments II-IV with lime-green colouration.

***Nanophyllium* Redtenbacher, 1906**

Type Species: *Nanophyllium pygmaeum* Redtenbacher, 1906 by original monotypy

Differentiation:

Female: Antennae with nine or ten segments; posteriormedial tubercle of the head capsule split into two points; mesopleura on their anterior end are notably wider than the prescutum anterior width; mesopleura always with prominent tubercles.

Male: Profemoral interior lobe, in both species groups with most often three small teeth; posteromedial tubercle of the head capsule split into two points; mesopleura on their anterior end are moderately wider than the prescutum anterior width; tegmina short, length never exceeding the posterior of the metathorax; alae radius splits into the first radial and the radial sector on the distal half of the wing and these two veins run separately to the wing margin without fusing to others; vomer long and slender with a single apical hook

Egg: Perfectly pentagonal in cross-section; no pinnae.

Fresh Nymph: (Only known for *asekiense*) slender abdomen; tibiae simple, but femoral lobes are more prominently formed with sharp margins; white striping on the meso- and metafemoral lobes, not fully crossing the leg (mostly just on the exterior lobe and femoral shaft); Abdominal segments II-IV with prominent green spots.

Supplementary Note 2

Keys to Phylliidae genera (adapted from Cumming et al.[12])

Key to females

1 Posteriormedial tubercle of the head capsule split into two lobes………………………………………….. ………………………………………………………………………*Nanophyllium* **Redtenbacher, 1906**

– Posteriormedial tubercle of the head capsule singular, not split into two lobes……....……………………**2**

2 Tegmina venation with the posterior cubitus split into an anterior cubitus (CuA), first posterior cubitus (CuP1), and second posterior cubitus (CuP2)……………………***Walaphyllium*** **Cumming et al., 2020**

– Tegmina cubitus venation simple (unsplit) or bifurcate (into an anterior cubitus (CuA) and posterior cubitus (CuP1) only)...**3**

3 Pro-, meso-, and metatibiae exterior with lobes; <u>and</u> tegmina with media and cubitus veins running side by side, touching throughout the majority of their lengths...............***Pulchriphyllium*** **Griffini, 1898**

– Pro-, meso-, and meatibiae simple, lacking exterior lobes (in most, but if exterior lobes are present partially only on the distal portion or very rarely fully spanning the shaft) then the tegmina media and cubitus veins are distinctly separated with several vein width distances between them throughout the length, not touching…………………..**4**

4 Prescutum spination along the sagittal plane with the middle or posterior spine most prominent, the anterior most is never most prominent…………………………………………………………………….**5**

– Prescutum sagittal plane either with: the spine on the anterior rim most prominent, a distinct anterior rim followed by granulation, or with spination of equal size from the anterior to the posterior……………………………………………..……..………..**6**

5 Posterior most spine of the prescutum is the most prominent; gonapophyses which are long, reaching to the apex of the anal abdominal segment; third antennomere with stridulantory file……….………… …...……………………………………….……………………***Pseudomicrophyllium*** **Cumming, 2017**

– Middle most spine along the prescutum is the largest spine and the posterior most spine is highly reduced; gonapophyses notably short, only slightly protruding from under the subgenital plate; third antennomere lacking stridulantory file….…………………………………***Microphyllium*** **Zompro, 2001**

6 Terminal antennomere as long as the preceding three to five segments combined……………………… ………….…………………………………………………………***Comptaphyllium*** **Cumming et al., 2019**

– Terminal antennomere as long as the preceding one or two segments combined...............................**7**

7 Tegmina with media and cubitus veins running side by side, touching throughout the majority of their lengths or fused throughout most of their length…..……………………………..***Chitoniscus*** **Stål, 1875**

– Tegmina with media and cubitus veins with significant spacing between them (several vein widths away) not touching…………………………....……………….…………………………………**8**

8 Third antennomere with the proximal end broad and often slightly recurved, making the segment a similar width throughout, or broader on the proximal end; fourth antennomere short and disk-like at least 3× wider than long and notably shorter than any of the following three segments, or rarely a similar length to the following segment, but still at least 2× as wide as long……………………………… ....***Cryptophyllium*** **Cumming et al., 2021**

– Third antennomere narrowest on the proximal end, broadening to the distal end; fourth antennomere typically as tall as wide and of a similar height to each of the following three segments length, not notably shorter...***Phyllium*** **Illiger, 1798**

Key to males

1 Posteriormedial tubercle of the head capsule split into two lobes……*Nanophyllium* **Redtenbacher, 1906**

– Posteriormedial tubercle of the head capsule singular, not split into two lobes………………………………**2**

2 Small (< 30.0 mm in length); protibiae lacking an interior lobe; restricted to the Philippines…...............**3**

– Medium to large (35.0 mm to > 80.0 mm); protibiae almost always with a half to fully developed interior lobe, or rarely highly reduced to a sliver on the proximal half only………………………………………**4**

3 Antennae short (only ca. the length of the outstretched front legs), with bead-like antennomeres that are no more than 2× longer than they are wide; profemoral interior lobes which are narrow and marked with three distinct teeth………...***Microphyllium*** **Zompro, 2001**

– Antennae notably longer than the outstretched front legs, with antennomeres 4–5× longer than wide; profemoral interior lobes reduced to only a single anterior spine………………………………………..***Pseudomicrophyllium*** **Cumming, 2017**

4 Prescutum stout, ca. 2× as wide as long……………….....................................***Chitoniscus*** **Stål, 1875**

– Prescutum as long as wide or notably longer than wide………..**5**

5 Vomer with two apical hooks……………….........................***Cryptophyllium*** **Cumming et al., 2021**

– Vomer with a single apical hook...**6**

6 Alae radial sector, media anterior, and media posterior veins fusing to the cubitus at different locations along the vein and running together to the wing margin…….................***Pulchriphyllium*** **Griffini, 1898**

– Alae radial sector, media anterior, and media posterior not fusing with the cubitus……………........**7**

7 Tegmina media vein splits into the anterior media vein (MA) and posterior media vein (MP) very early on, immediately or at most 1/4 of the way through the wing length and they run unbranched and subparallel through the wing length; protibial interior lobe not reaching from end to end of the shaft, only restricted to the proximal 1⁄2 to 2⁄3 but never more; a head capsule with clearly defined nodes arranged in evenly spaced patterns......................................***Comptaphyllium*** **Cumming et al., 2019**

– Tegmina media vein running unbranched for the first 1⁄3 to 2⁄5 of the wing length, and then branching with either a single short media posterior running to the wing margin or two short media posteriors branching from the notably longer media anterior and running to the margin; protibial interior lobe variable, either fully spanning the full length or only 1⁄2 of the length; head capsule at most with random granulation but frequently bare...**8**

8 Abdominal shape rectangular, with segments V and VI with fully parallel-sided margins (segments IV and VII with only half parallel-sided and the remainder curved)………………………………………..***Walaphyllium*** **Cumming et al., 2020**

– Abdominal shape variable, either spade-shaped (with the margins of V parallel or strongly converging and segment VI strongly converging), ovular (with margins expanding and then contracting, no segments parallel-sided), thin and slender with converging margins, bell-shaped (with margins expanding until segment VI then strongly converging) or boxy with only segment V parallel-sided (segments IV and VI only partially parallel-sided, the remainder rounded……..***Phyllium*** **Illiger, 1798**

Key to eggs

Unknown for *Microphyllium* Zompro, 2001

1 Surface lacking pinnae, instead porous and brittle, or granular………...**2**

– Surface with pinnae (moss, rope, or feather-like)..**6**

2 Capsule longer than wide, with flat surfaces……………………………………………………………**3**

– Capsule spherical, no flat surfaces………..............................***Pseudomicrophyllium* Cumming, 2017**

3 Pentagonal in cross-section with all surfaces equally wide……….***Nanophyllium* Redtenbacher, 1906**

– Cross-section with lateral surfaces or dorsal surface notably broader than others, not perfectly pentagonal in cross-section……………………………………………………………………………...**4**

4 Lateral margins fanned out into distinct fins with an operculum which is typically longer than wide (but not always), or if the capsule fins are reduced (not prominently protruding), the egg in cross-section is distinctly triangular (not pentagonal or rectangular), with the dorsal surface notable broader than the other surfaces..***Pulchriphyllium* Griffini, 1898**

– Capsule boxy and rectangular without distinct fins and the operculum is always notable wider than long (length ca. 1⁄2 the greatest width)...**5**

5 Eggs medium to large, 5.0–7.0 mm long; Australia and New Guinea only…………………………………
....***Walaphyllium* Cumming et al., 2020**

– Eggs small, ca. 3.0 mm long; Fiji and New Caledonia only................................***Chitoniscus* Stål, 1875**

6 Pinnae short and moss-like over the entire capsule..................***Cryptophyllium* Cumming et al., 2021**

– Pinnae long and feather or rope like...**7**

7 Operculum with a row of pinnae along the sagittal plane, not pinnae encircling the margin of the operculum..***Comptaphyllium* Cumming et al., 2019**

– Operculum with pinnae encircling the margin, not along the sagittal plane………………..................
....***Phyllium* Illiger, 1798**

Supplementary References

1. Cumming, R. T., Baker, E., Le Tirant, S. & Marshall, J. On the Phyllium Illiger, 1798 of Palawan (Philippines), with description of a new species (Phasmida: Phylliidae). *Faunitaxys* **8**, 1–9 (2020).
2. Cumming, R. T., Le Tirant, S. & Hennemann, F. H. Review of the Phyllium Illiger, 1798 of Wallacea, with description of a new subspecies from Morotai Island (Phasmatodea: Phylliidae: Phylliinae). *Faunitaxys* **7**, (2019).
3. Cumming, R. T., Bank, S., Le Tirant, S. & Bradler, S. Notes on the leaf insects of the genus Phyllium of Sumatra and Java, Indonesia, including the description of two new species with purple coxae (Phasmatodea, Phylliidae). *Zookeys* **913**, 89–126 (2020).
4. Cumming, R. T., Le Tirant, S. & Teemsma, S. N. On the Phyllium of Peninsular Malaysia and Sumatra, Indonesia, with range expansions for currently known species, description of the previously unknown Phyllium (Pu.) abdulfatahi Seow-Choen female, and description of the new species Phyllium (Ph.) rubrum . *Faunitaxys* **6**, 1–21 (2018).
5. Redtenbacher, J. Phasmidae: Areolatae. in *Die Insektenfamilie der Phasmiden, Volume 1* 1–180 (Wilhelm Engelmann, Leipzig, 1906).
6. Günther, K. Über die taxonomische Gliederung und geographische Verbreitung der Insektenordnung der Phasmatodea. *Beiträge zur Entomol.* **3**, 541–563 (1953).
7. Griffini, A. Sur les Phyllidae. *Extr. des Misc. Entomol.* **6**, 8–12 (1898).
8. Hennemann, F. H., Conle, O. V., Gottardo, M. & Bresseel, J. On certain species of the genus Phyllium Illiger, 1798, with proposals for an intra-generic systematization and the descriptions of five new species from the Philippines and Palawan (Phasmatodea: Phylliidae: Phylliinae: Phylliini). *Zootaxa* **2322**, 1–83 (2009).
9. Cumming, R. T., Le Tirant, S. & Hennemann, F. H. A new leaf insect from Obi Island (Wallacea, Indonesia) and description of a new subgenus within Phyllium Illiger, 1798 (Phasmatodea: Phylliidae: Phylliinae). *Faunitaxys* **7**, 1–9 (2019).
10. Cumming, R. T., Thurman, J. H., Youngdale, S. & Le Tirant, S. Walaphyllium subgen. nov., the dancing leaf insects from Australia and Papua New Guinea with description of a new species (Phasmatodea, Phylliidae). *Zookeys* **939**, 1–28 (2020).
11. Brock, P. D., Büscher, T. H. & Baker, E. SF Phasmida: Phasmida Species File (version 5.0). in *Species 2000 & ITIS Catalogue of Life, 2020-09-01 Beta* (eds. Roskov, Y. & Al., E.) (Naturalis, Leiden, 2020).
12. Cumming, R. T. *et al.* Cryptophyllium, the hidden leaf insects – descriptions of a new leaf insect genus and thirteen species from the former celebicum species group (Phasmatodea, Phylliidae). *Zookeys* **1018**, 1–179 (2021).
13. Buckley, T. R., Attanayake, D. & Bradler, S. Extreme convergence in stick insect evolution: Phylogenetic placement of the Lord Howe Island tree lobster. *Proc. R. Soc. B Biol. Sci.* **276**, 1055–1062 (2009).
14. Bradler, S., Cliquennois, N. & Buckley, T. R. Single origin of the Mascarene stick insects: Ancient radiation on sunken islands? *BMC Evol. Biol.* **15**, 196 (2015).
15. Robertson, J. A., Bradler, S. & Whiting, M. F. Evolution of oviposition techniques in stick and leaf insects (Phasmatodea). *Front. Ecol. Evol.* **6**, 216 (2018).
16. Forni, G. *et al.* Macroevolutionary analyses provide new evidences of phasmids wings evolution as a reversible process. *bioRxiv* (2020) doi:10.1101/2020.10.14.336354.

Supplementary Figures

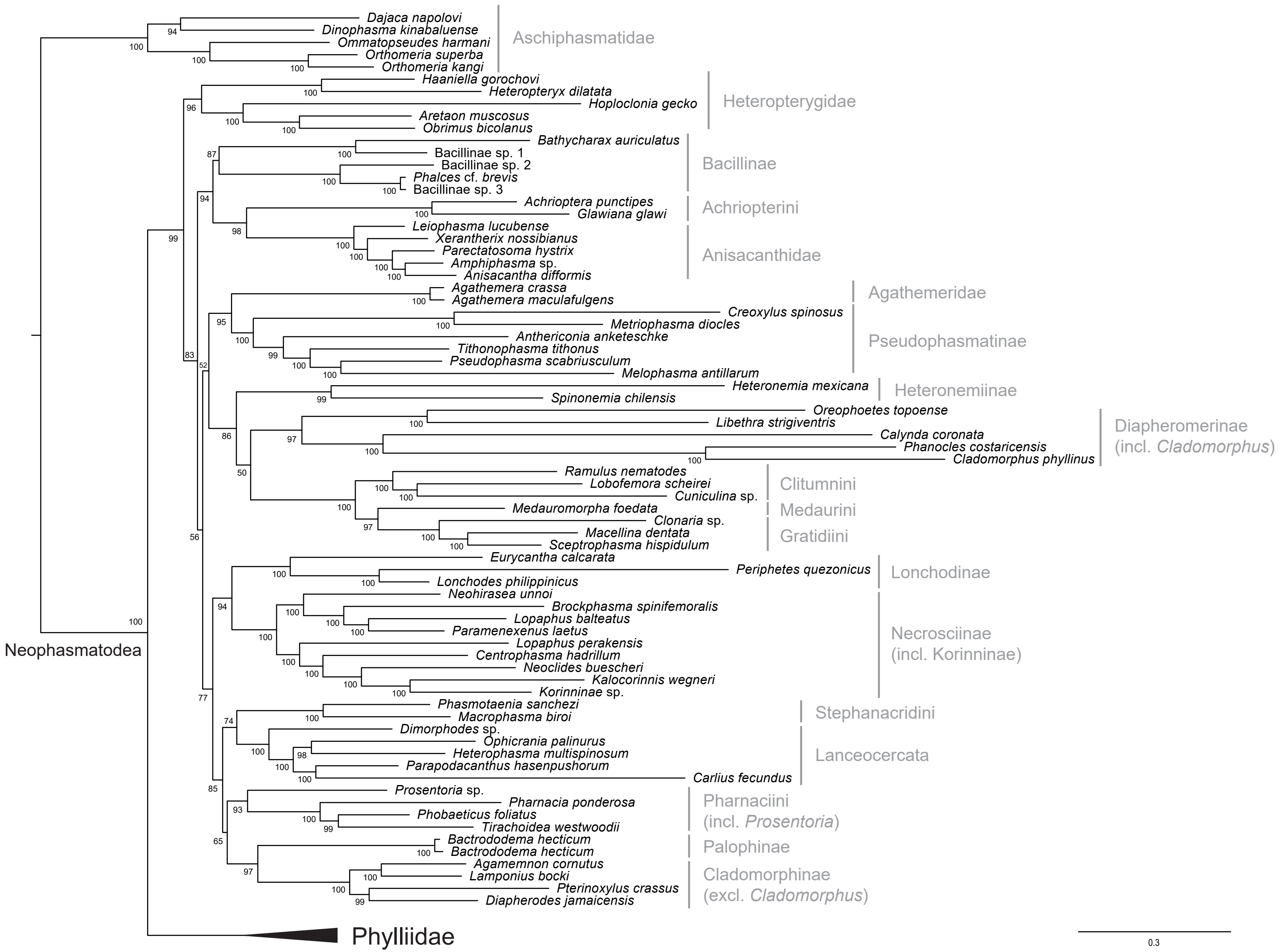

Supplementary Figure 1. The best-scoring ML tree (outgroup taxa to Figure 2) depicting the phylogenetic hypothesis for Euphasmatodea. The tree was rooting with Aschiphasmatidae and UFBoot support values are placed at each node.

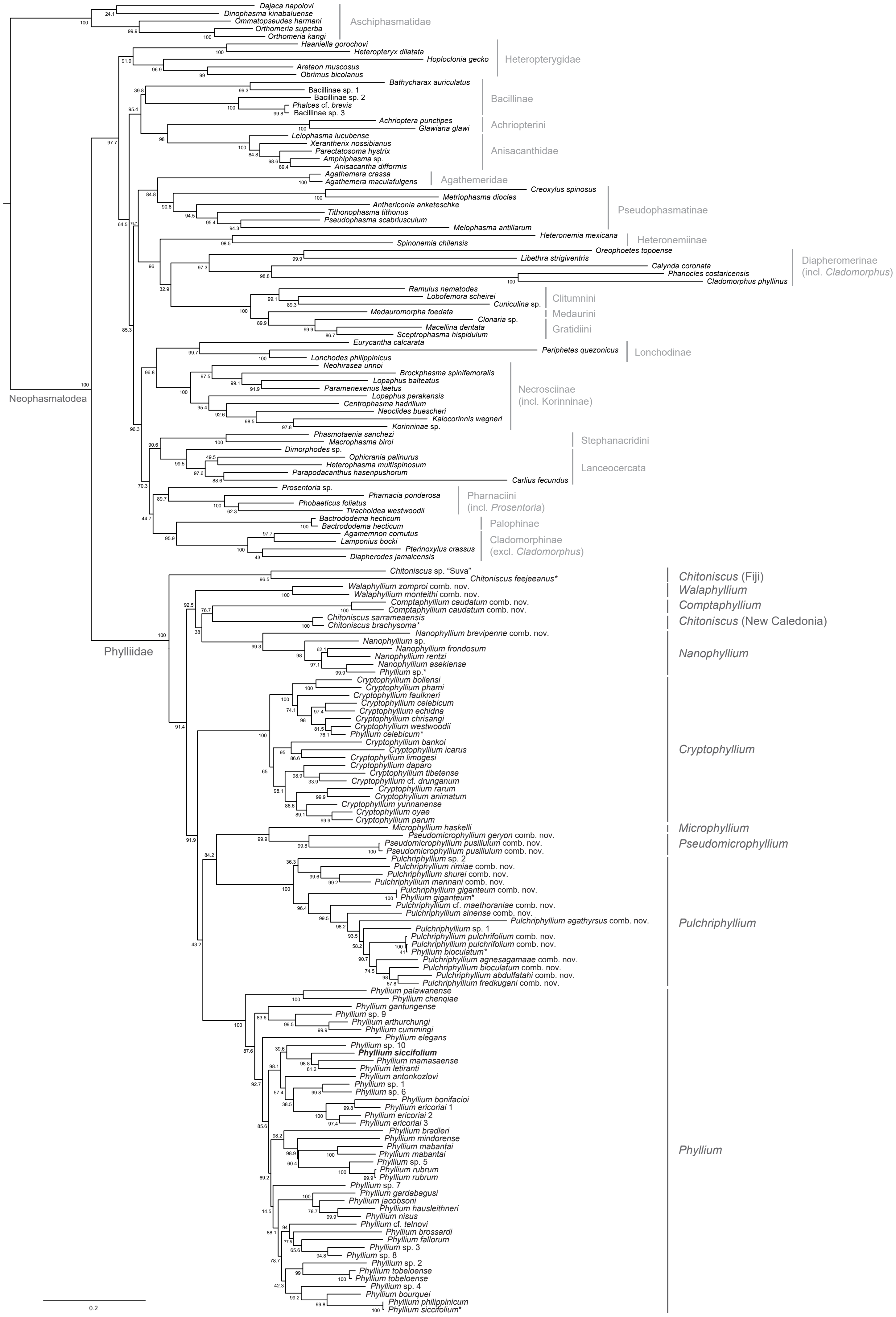

Supplementary Figure 2. The best-scoring ML tree (as in Figure 2 and Supplementary Figure 1) with node support values derived from the Shimodaira-Hasegawa-like approximate likelihood ratio test (SH-aLRT).

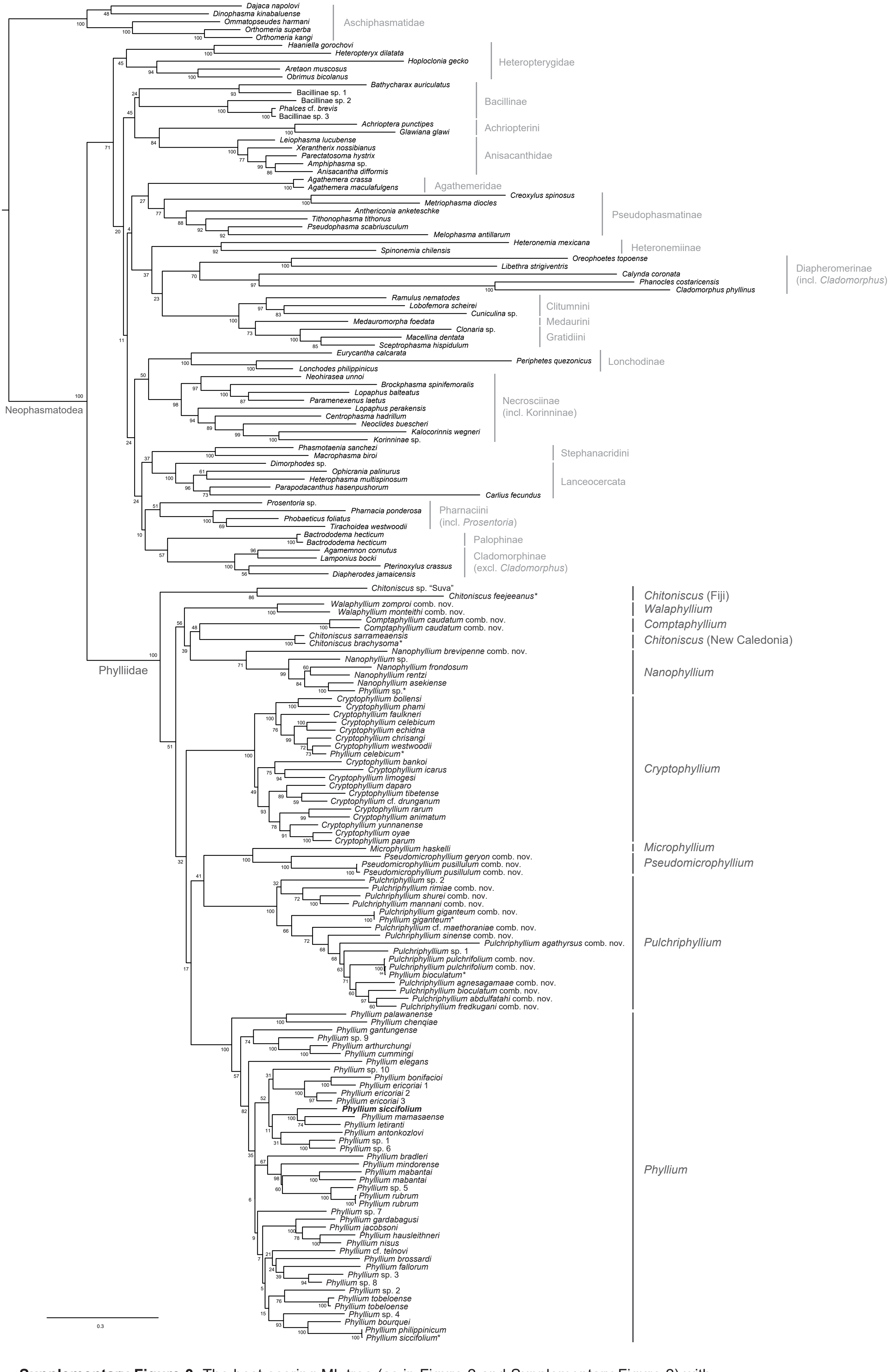

Supplementary Figure 3. The best-scoring ML tree (as in Figure 2 and Supplementary Figure 2) with nonparametric standard bootstrap (BS) node support values.

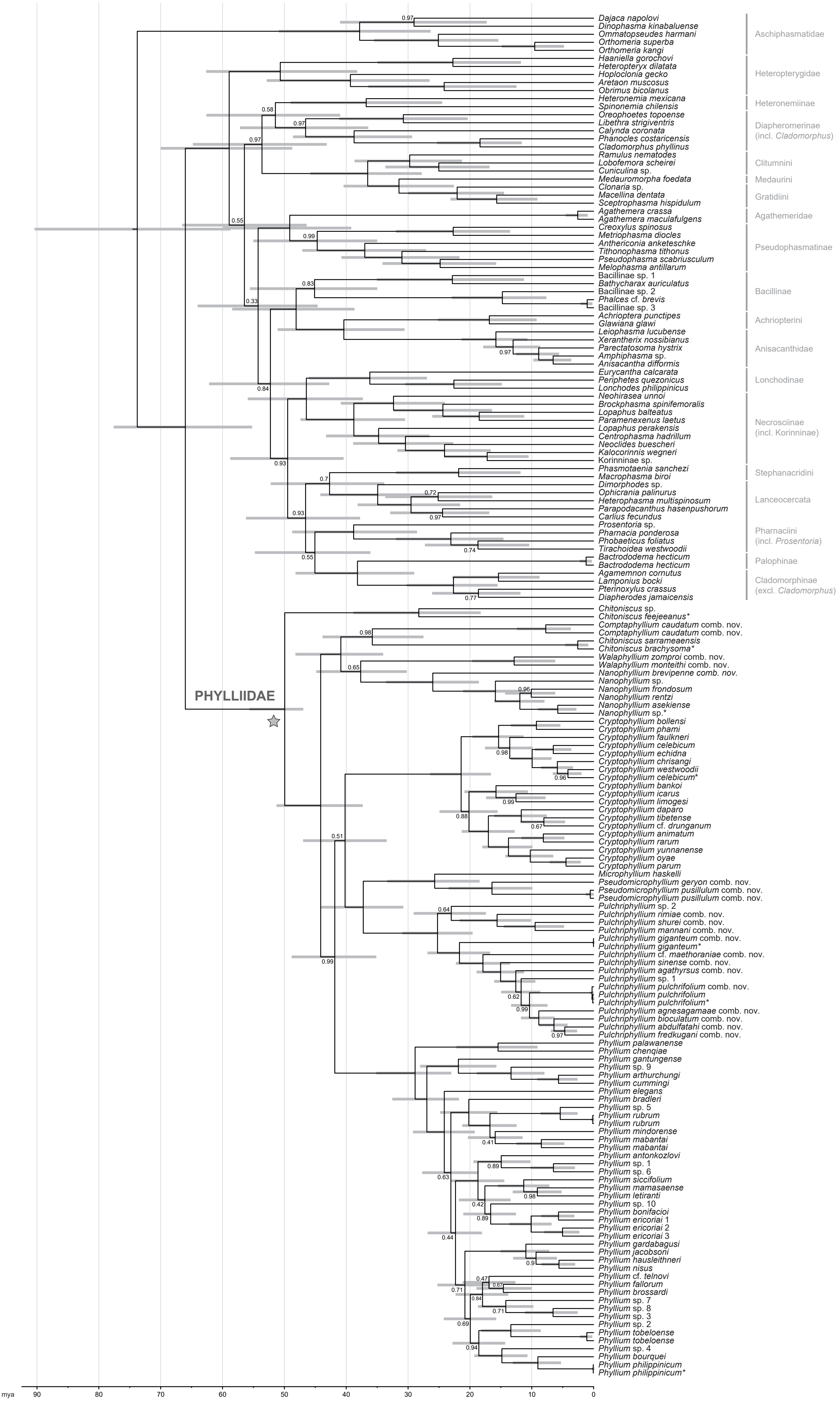

Supplementary Figure 4. The complete BI time tree (as partially used in Figure 5) based on the fossil calibration using *Eophyllium messelense*. The node on which the calibration was used is highlighted by a star. The 95% credibility intervals and nodal support of <1 PP is given at the node.

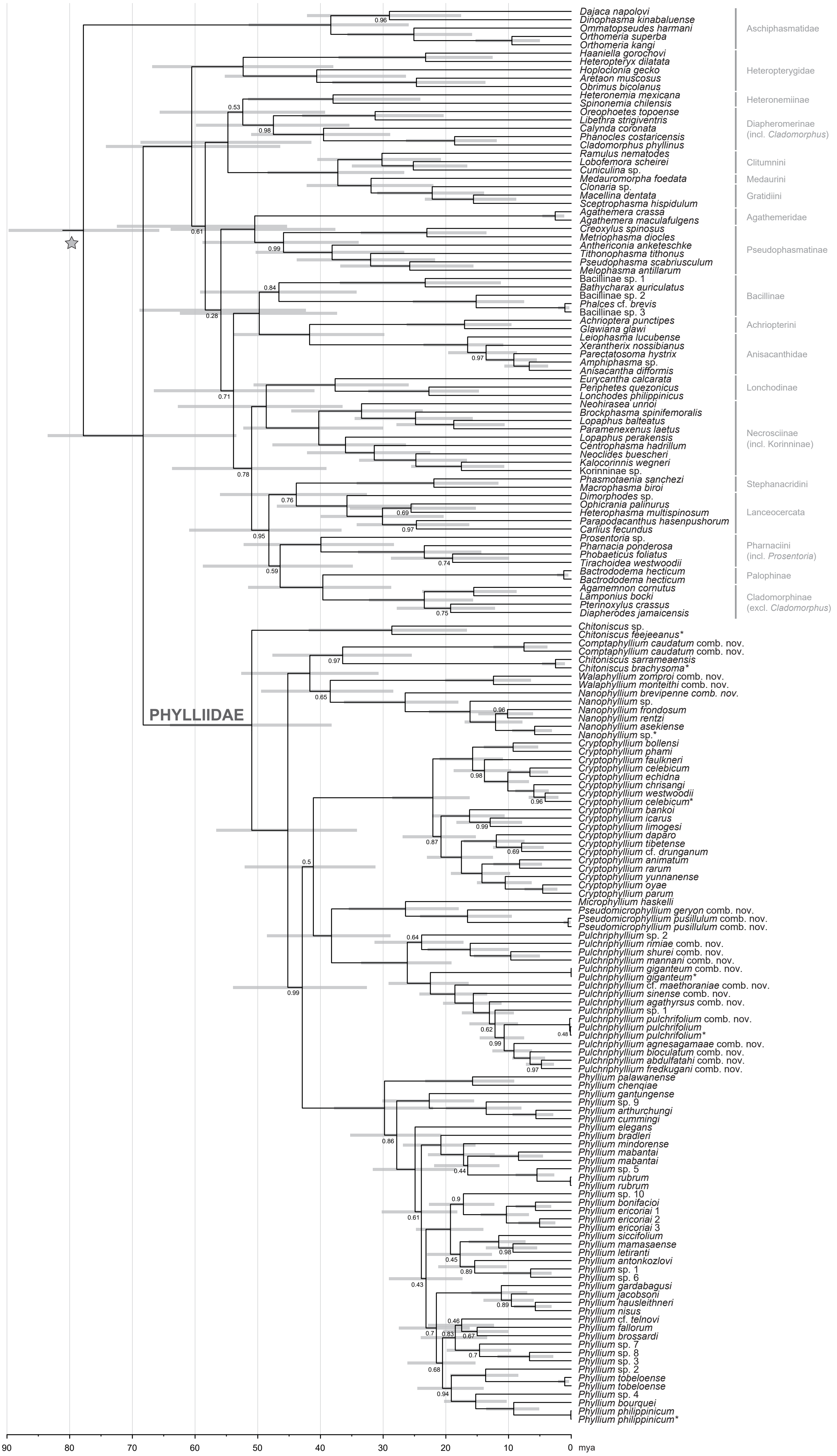

Supplementary Figure 5. BI tree run under the same settings as Supplementary Figure 4 but without fossil calibration. Instead, a secondary calibration for the root (Euphasmatodea) was used (highlighted by a star). The 95% credibility intervals and nodal support of <1 PP is given at the node.

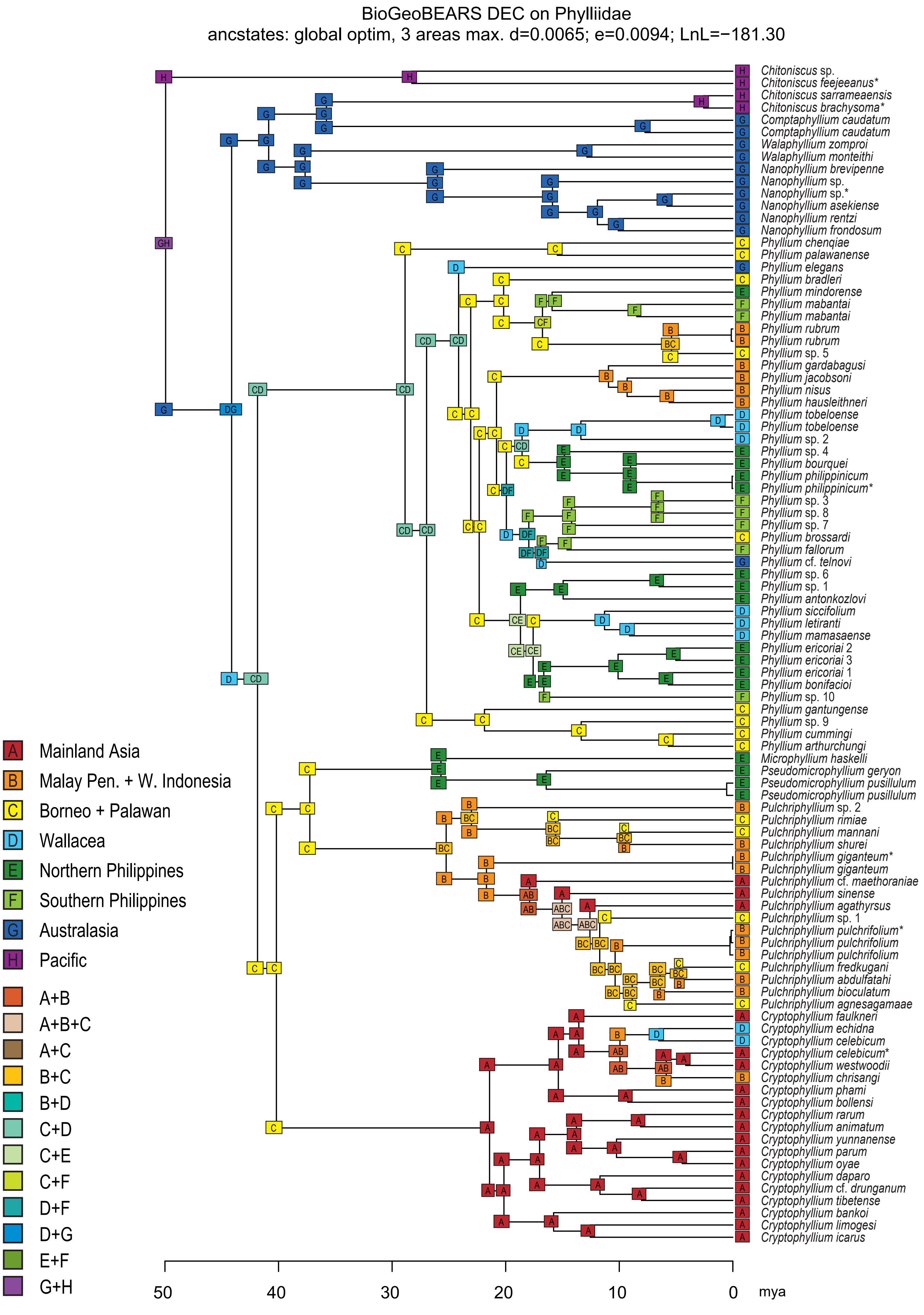

Supplementary Figure 6a. Results of ancestral range estimation using the biogeographic model DEC in BioGeoBEARS.

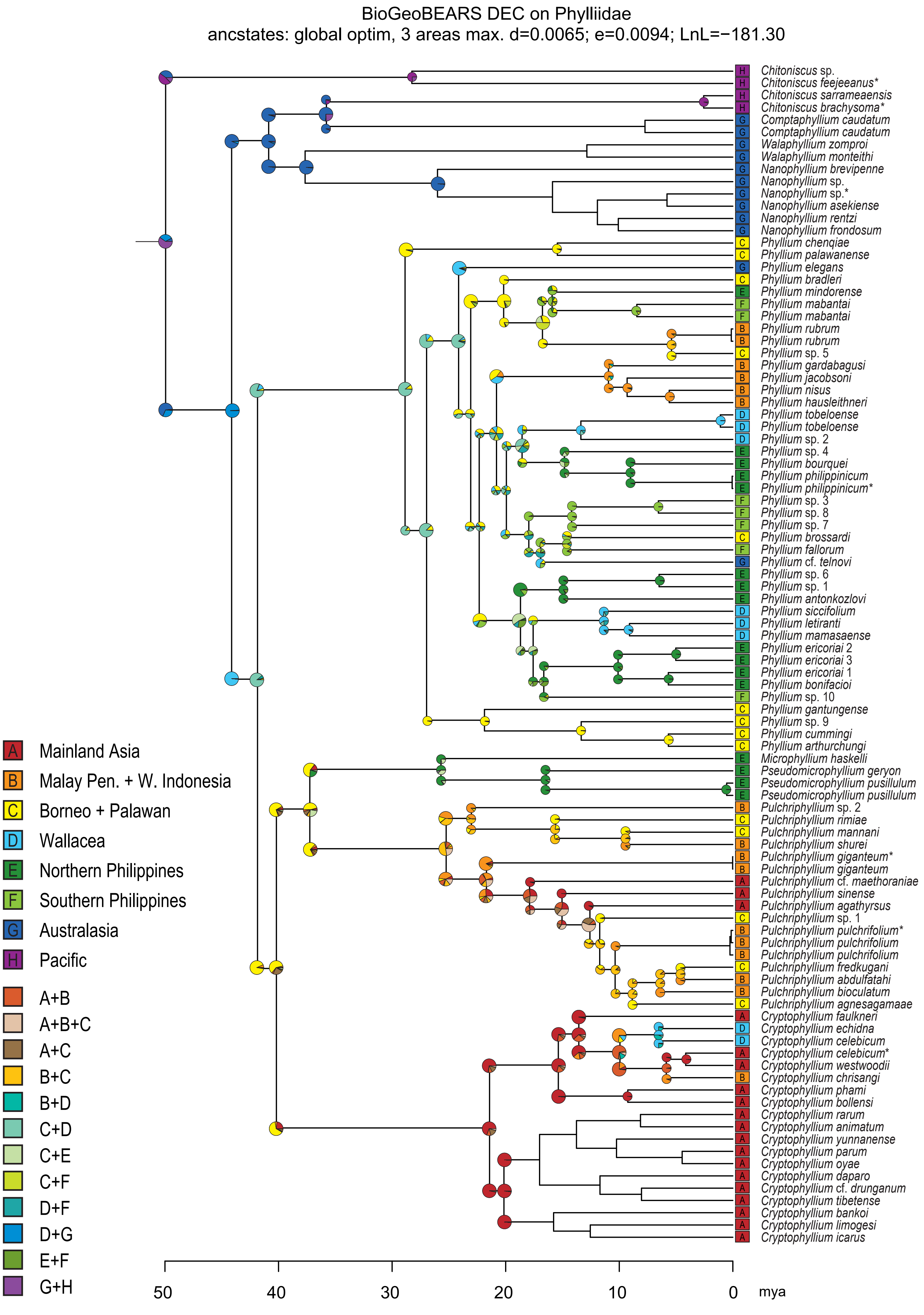

Supplementary Figure 6b. Results of ancestral range estimation using the biogeographic model DEC in BioGeoBEARS with probability pie charts at each node.

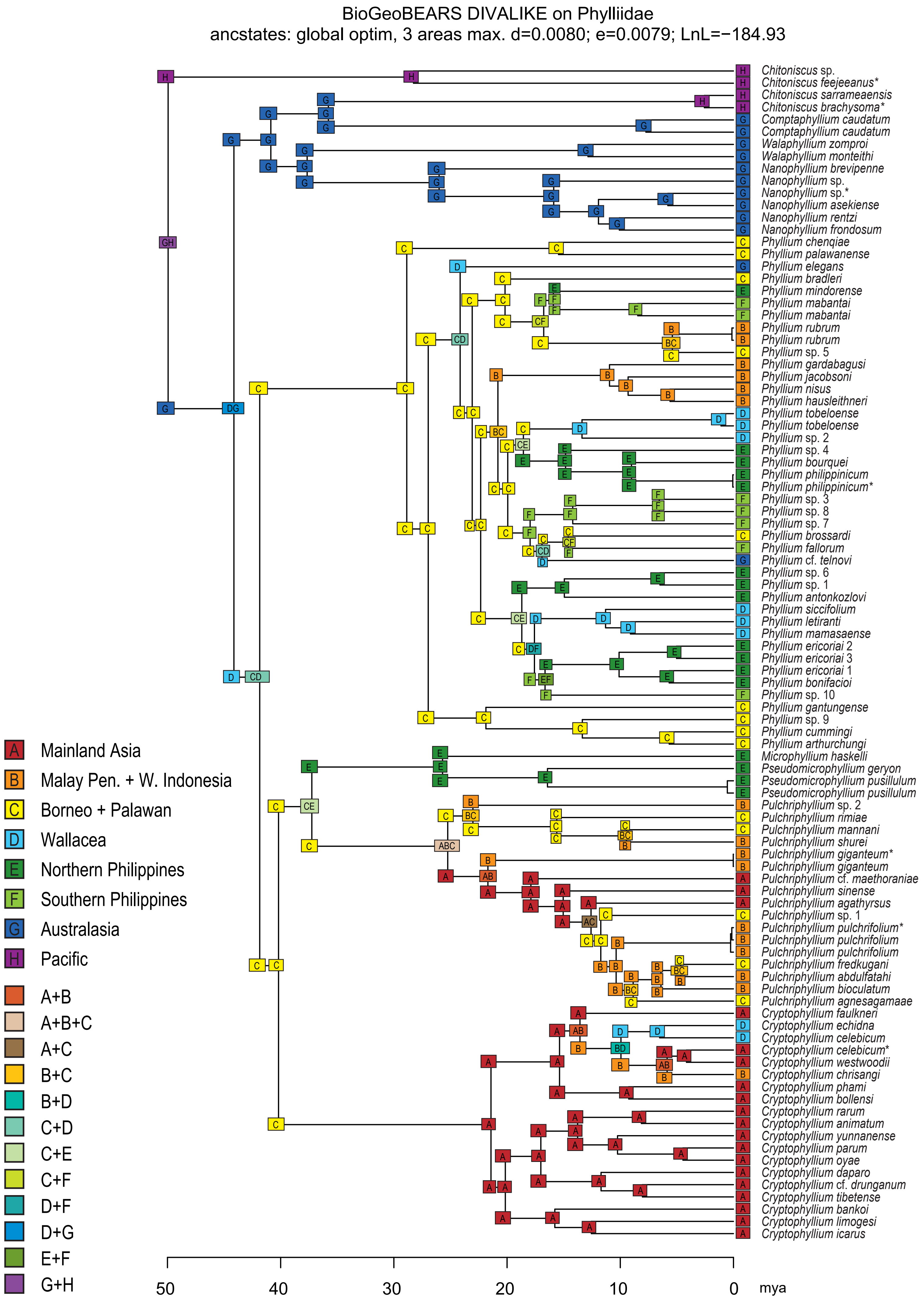

Supplementary Figure 6c. Results of ancestral range estimation using the biogeographic model DIVALIKE in BioGeoBEARS.

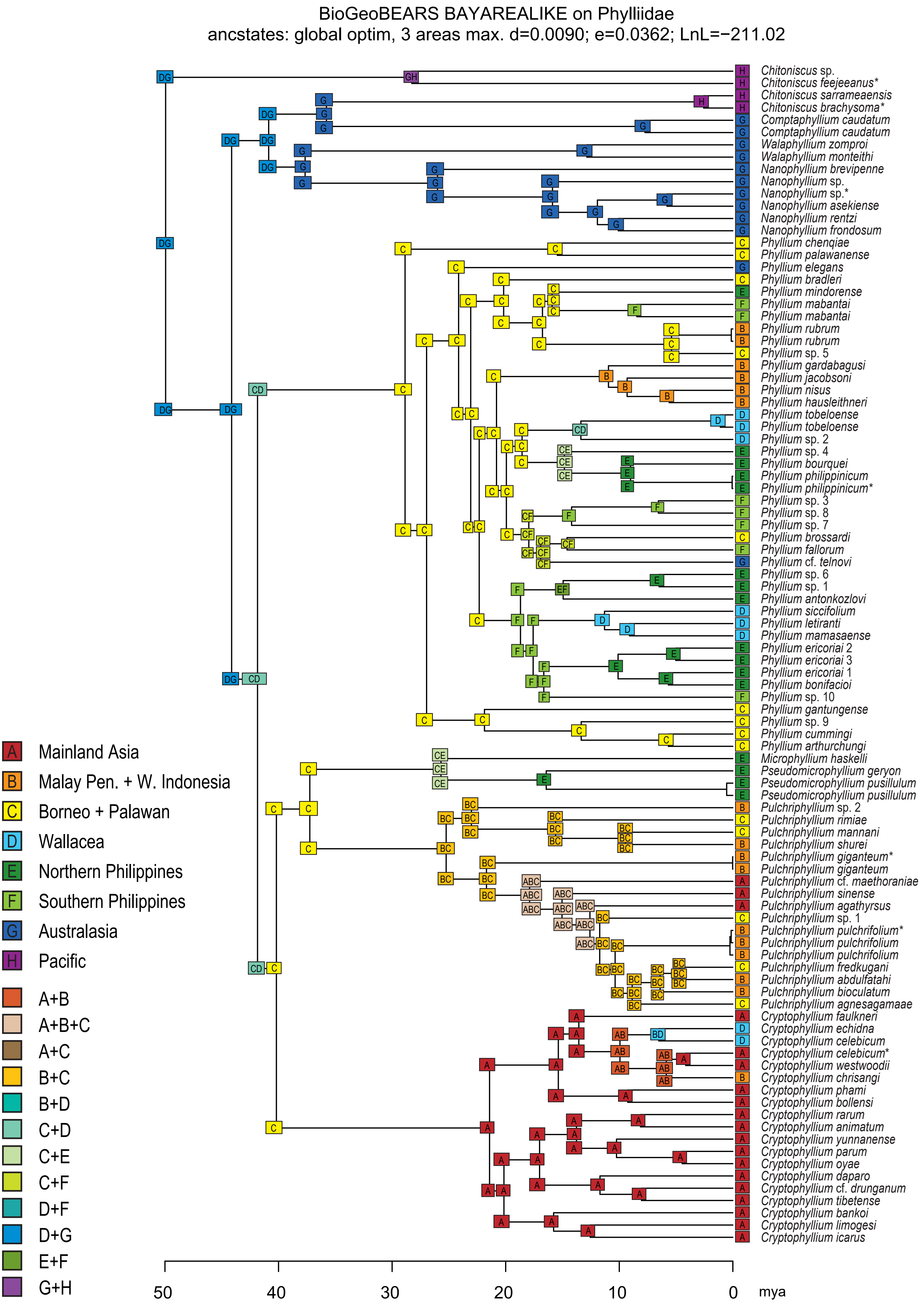

Supplementary Figure 6d. Results of ancestral range estimation using the biogeographic model BAYAREALIKE in BioGeoBEARS.
